# A dual cardiomyocyte reporter model derived from human pluripotent stem cells

**DOI:** 10.1186/s13287-021-02341-6

**Published:** 2021-05-29

**Authors:** Yuqian Jiang, Xiaoping Bao, Xiaojun Lance Lian

**Affiliations:** 1 Department of Biomedical Engineering, Pennsylvania State University, University Park, PA 16802 USA; 2 Davidson School of Chemical Engineering, Purdue University, West Lafayette, IN 47907 USA; 3 Department of Biology, Pennsylvania State University, University Park, PA 16802 USA; 4 Huck Institutes of the Life Sciences, Pennsylvania State University, University Park, PA 16802 USA

**Keywords:** Atrial or ventricular cardiomyocytes, Human pluripotent stem cells, ASAP2f indicator, Alpha MHC promoter, Dual reporter

## Abstract

Cardiovascular diseases (CVD) remain the leading cause of death in the USA. Cardiomyocytes (CMs) derived from human pluripotent stem cells (hPSCs) provide a valuable cell source for regenerative therapy, disease modeling, and drug screening. Here, we established a hPSC line integrated with a mCherry fluorescent protein driven by the alpha myosin heavy chain (aMHC) promoter, which could be used to purify CMs based on the aMHC promoter activity in these cells. Combined with a fluorescent voltage indicator, ASAP2f, we achieved a dual reporter CM platform, which enables purification and characterization of CM subtypes and holds great potential for disease modeling and drug discovery of CVD.

**Supplementary Information:**

The online version contains supplementary material available at 10.1186/s13287-021-02341-6.

## Introduction

Cardiovascular diseases (CVD) claim millions of lives per year and pose a severe threat to economics and human health. What is worse, the total number of annual fatalities caused by CVD worldwide is projected to increase [1]. Transplantation of cadaveric heart donors emerges as a potential therapy for CVD and has been performed since the first case in 1967. So far, due to advances in knowledge of preventing rejection and infection post-heart transplantation, the survival rate has been distinctly elevated. Nevertheless, despite more refined criteria of recipient-donor selection and improved regulation of allocation or prioritization of donor resources, shortage of donor hearts remains a primary limitation, leading to a lengthy waiting list for CVD patients [2].

Functional CMs derived from human pluripotent stem cells (hPSCs) would provide an unprecedented cell source for disease modeling, drug discovery, and cell transplantation therapy for CVD patients [3]. Despite the high efficiency of CM differentiation with recent advances [4, 5], the resulting cultures are mixed with different CM subtypes, including atrial, ventricular, and a small number of pacemaker CMs, which may lead to potential problems such as influencing in vitro disease modeling outcomes and altering in vivo cell function after transplantation. Therefore, it is critical to generate CM models composed of homogenous subtypes. Early in 2003, Mummery et al. cocultured hPSCs with mouse visceral endoderm-like cells, generating enriched ventricular-like CMs (around 85%). This report demonstrated the possibility of inducing biased CM subtypes and inspired the following researches [6]. Zhang and colleagues investigated the role of Noggin and retinoid signaling in the specification of CM subtypes, which led to the increased proportions of atrial or ventricular-like CM populations [7]. Likewise, by manipulating the retinoid pathway, the Keller group reported the dependence of atrial/ventricular specification on early mesoderm patterning and further identified surface markers RALDH2 and CD235a to efficiently distinguish atrial and ventricular subtypes respectively [8]. Halloin et al. reported a chemically defined and xeno-free protocol for suspension culture of ventricular-like CMs with >90% purity by controlling the WNT pathway [9]. Zhu and colleagues proved that inhibition of neuregulin (NRG)-1β/ErbB signaling enhanced the population of nodal-like CMs [10]. The Cho group pointed out that overexpression of gene *SHOX2* was sufficient to increase hPSC-derived cardiac pacemaker cells [11]. Schweizer et al. reported a protocol to selectively differentiate hPSCs into CMs with nodal-type characteristics, which relies on co-culture with visceral endoderm-like cells and subsequent culture in a serum-enriched medium [12]. The Keller group described a transgene-independent method to generate sinoatrial node (SAN)-like pacemaker cells, which were identified as NKX2-5-negative CMs expressing SAN markers and displaying expected electrophysiological properties [13]. However, all these protocols did not support dynamic cell monitoring during differentiation [8].

Fluorescent stem cell reporters provide a convenient and efficient method to purify expected CM subtypes. However, previous fluorescent reporters for CM studies relied on the integration of fluorescent proteins into early CM marker like *NKX2-5* or pan-CM marker like *NCX1*. These approaches generated a mixed population of SA node, ventricular CMs, or atrial CMs [14, 15]. Recently, the Wu group generated a TBX5^Clover2^ and NKX2-5^TagRFP^ double reporter cell line, which facilitated the isolation of four distinct cardiac subpopulations [16]. Specifically, TBX5+NKX2-5+ cells sorted at the second week of hPSC-CM differentiation gave rise to ventricular-like CMs with 93% purity and TBX5-NKX2-5+ cells held the potential to derive atrial-like CMs with 90% purity, which provided useful cell sources for precise drug testing. This study also utilized the fluorescent voltage sensor ASAP2 to analyze action potentials of CM subtypes. Nevertheless, the overlapping excitation and emission wavelengths of Clover2 (excitation 505nm; emission 515nm) with ASAP2 (excitation 488nm; emission 507nm) may introduce errors in fluorescence-based functional characterization in CMs. Here, we established a hPSC line integrated with aMHC promoter-driven mCherry reporter protein (excitation 587nm; emission 610nm), enabling the visualization and separation of derived atrial CMs. Combined with the advanced fluorescent voltage indicator, ASAP2f, we achieved a dual reporter hPSC-CM platform that enables both separation and microscope-based characterization of CM subtypes.

## Results

### Generation of aMHC-mCherry hPSC line

**Fig. 1:**
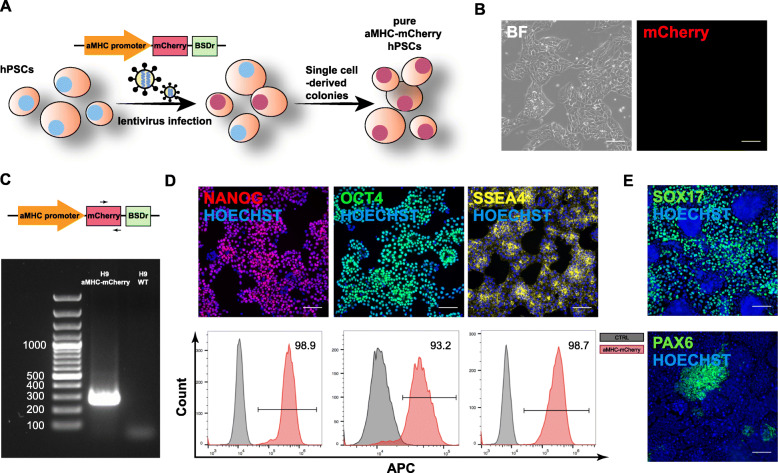
Generation of aMHC-mCherry H9 reporter line. **a** Diagram of the proposed strategy for reporter line derivation. Construct expressing aMHC promoter-driven mCherry and blasticidin-resistant (BSDr) genes was designed and delivered into hPSCs via lentivirus, followed by drug selection and single-cell colony derivation. **b** Representative brightfield (BF) and mCherry images of live aMHC-mCherry H9 cells. **c** PCR genotyping of hPSC clones after drug selection is shown. Forward and reverse primers were designed within mCherry coding sequencing, and wild-type (WT) H9 cells were used as a negative control. **d** Representative immunostaining and flow cytometry results of NANOG, OCT4, and SSEA4 are shown. **e** Representative immunostaining images of SOX17 and PAX6 for endodermal and ectodermal differentiation, respectively, were shown. Scale bars, 100 μm

Since aMHC is predominantly expressed in the human atrium instead of the ventricle [17], we designed the aMHC-mCherry construct to report atrial hPSC-CMs. Lentiviruses carrying the aMHC-mCherry construct were used to infect H9 hPSCs, and single cell-derived reporter H9 cells were obtained after drug selection (Fig. 1a). The resulting cells presented typical tightly packed and domed colony-like morphology of undifferentiated hPSCs with refractive edges (Fig. 1b). Genotyping with mCherry-targeting primers demonstrated the successful integration of the fluorescent mCherry reporter (Fig. 1c). Importantly, no mCherry fluorescence was observed in the undifferentiated hPSCs, indicating there was no leakage of mCherry signal (Fig. 1b). Immunostaining and flow cytometry analysis of pluripotency markers, including NANOG, OCT4, and SSEA4, demonstrated that engineered cells retained pluripotent status (Fig. 1d), which was further confirmed by their ability to differentiate into SOX17+ endoderm and PAX6+ ectoderm (Fig. 1e). No mCherry signal was observed during endoderm and ectoderm differentiation, indicating the lineage-specific activity of aMHC-mCherry. Mycoplasma test was performed routinely to ensure engineered reporter cells were not mycoplasma contaminated (Fig. S1).

### Characterization of aMHC-mCherry CMs

**Fig. 2:**
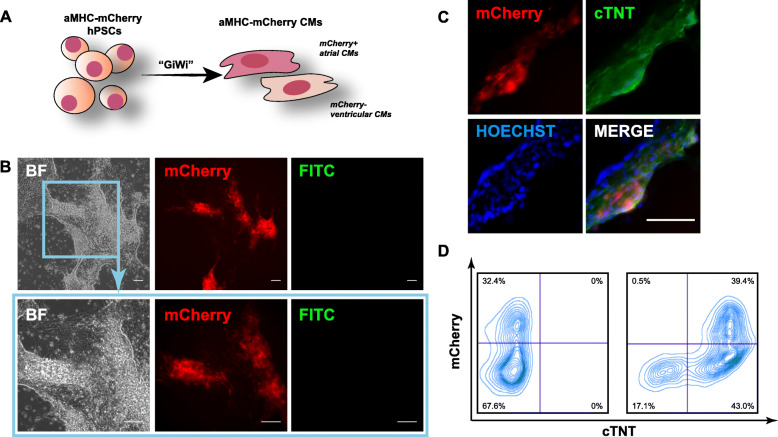
Cardiomyocyte (CM) derivation and subtype reporting of aMHC-mCherry hPSCs. **a** Diagram of CM subtype derivation, where aMHC-mCherry labels atrial CMs. **b** Representative brightfield (BF), mCherry, and FITC (empty channel) images of live aMHC-mCherry CMs on day 11. **c** Representative images of day 12 CMs stained with pan-CM marker cTNT. **d** Flow cytometry analysis of aMHC-mCherry CMs with (right) or without (left) cTNT staining on day 12. Scale bars, 100 μm

We next investigated the reporting function of the resulting aMHC-mCherry hPSCs during CM differentiation with our previously developed GiWi protocol [5] (Fig. 2a). Spontaneously beating CMs were successfully generated, and strong mCherry signal was observed (Fig. 2b; Video S1-S2), confirming the reporting function of aMHC-mCherry. Overlapping expression of mCherry with cardiac troponin T (cTNT) via both immunostaining and flow cytometry analysis (Fig. 2c, d) successfully demonstrated their cardiac-specific reporting and indicated the presence of another CM population that only expresses cTNT, but not mCherry. Based on that fact that aMHC is expressed predominantly in atria and it is distinct from the beta-MHC isoform that mainly locates in ventricles [17], the cTNT+mCherry+ cells we observed were atrial CMs, and the majority of cTNT+mCherry− cells were ventricular CMs. To further investigate the correlation between aMHC-mCherry signals and CM subtypes, we stained generated day 30 (D30) CMs with MLC2v and MLC2a antibodies, which specifically targeted ventricular CMs or atrial CMs, respectively. Based on images, we observed co-localization of mCherry signals with MLC2a+ CMs. MLC2v+ CMs, however, were mCherry negative (Fig. S2A, B), further supporting our results in flow cytometry. Therefore, this aMHC-mcherry reporter hPSC line possesses the potential to track atrial CM subpopulations during CM differentiation. When combined with surface cell marker, such as the pan-CM marker SIRPA, ventricular-enriched population (SIRPA+mCherry−) could also be easily isolated [18].

### Dual reporter platform

**Fig. 3:**
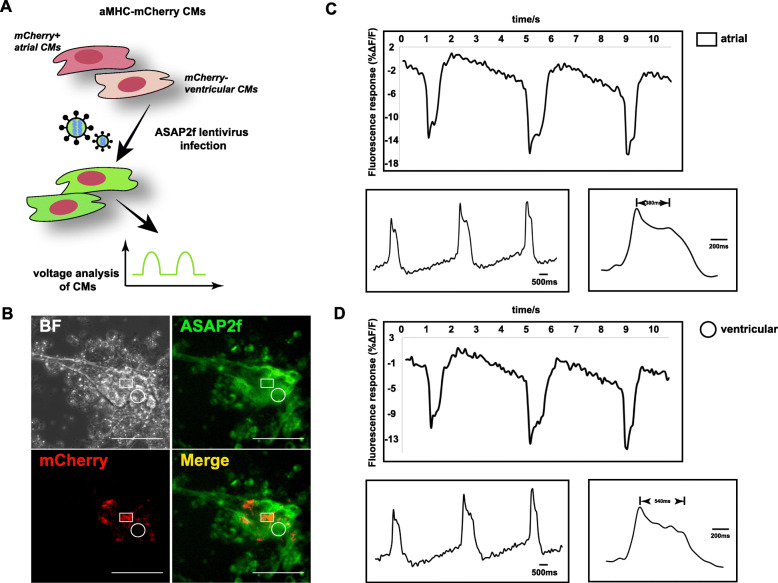
Generation of a dual aMHC-mCherry and ASAP2f voltage reporter CM model. **a** Diagram of the proposed strategy for combining aMHC-mCherry reporter with ASAP2f indicator. **b** Representative brightfield (BF), ASAP2f, and mCherry images of day 29 live CMs derived from dual reporter hPSCs. ASAP2f fluorescence signal analysis of atrial- (**c**) and ventricular-like (**d**) CMs at indicated locations illustrated in **b** was shown. Rectangular spot represents the mCherry+ASAP2f+ region, while circle spot indicates the mCherry-ASAP2f+ region. Scale bars, 100 μm

ASAP2f emerges as a sensitive voltage indicator to assess the performance and function of CMs, neurons, and other cell types with ion flux fluctuation [19, 20]. Before the development of these genetically encoded voltage indicators (GEVI), voltage-sensitive dyes were widely used for cellular imaging due to their brightness, photostability, and fast kinetics [21–24]. Nevertheless, there are concerns that the addition of these dye molecules can alter the electrical properties of the plasma membrane, thus distorting its normal behavior or slowing down action potential conduction. In addition, their substantial toxicity and dye-specific pharmacological side effects have also been reported, which remain challenges for using these chemogenic voltage dyes for cell imaging [25]. In comparison, GEVI show less risk of photobleaching or cell toxicity and have the advantage on tissue-specific imaging, which have been widely used for cell characterization. Here, by combining the aMHC-mCherry reporter with ASAP2f (Fig. 3a), we developed a dual-reporter hPSC platform for real-time live-cell visualization and functional analysis of hPSC-derived CMs during differentiation. To achieve this, the ASAP2f coding sequence was cloned into a lentiviral backbone and packaged to produce lentiviruses for hPSC infection. The majority of infected cells presented GFP fluorescence on the cell membrane (Fig. S3A), confirming the successful integration of ASAP2f in hPSCs while retaining strong expression of pluripotent marker (Fig. S3B). We next assessed the fluorescence fluctuation of ASAP2f, which could recapitulate the cell membrane potential by reporting voltage changes, in both atrial and ventricular CMs (Fig. 3b–d; Video S3, S4-S5). Changes in fluorescence intensity were recorded and used to reflect the voltage changes, presenting typical shapes of cardiac action potential with stages of rapid depolarization, initial repolarization, plateau, rapid repolarization, and resting potential. Interestingly, as compared to mCherry-negative regions, action potential shapes in mCherry-positive regions showed shorter duration of plateau stage, which was consistent with reported differences in action potentials of atrial and ventricular CMs via patch-clamp analysis [26] (Fig. 3c, d), underscoring the sensitivity and specificity of this dual reporter platform. Moreover, we tested isoprenaline, a drug that has been demonstrated to accelerate the heat beating [27], in the generated CMs from our reporter cell line. Consistent with previous observations, isoprenaline significantly increased the frequency of CM contraction in a dose-dependent manner (Fig. S4), further supporting the utility of this ASAP2f reporter design.

## Conclusions

In this study, we generated a stable hPSC line with mCherry reporter driven by aMHC promoter, enabling the visualization and separation of atrial CM subpopulations from hPSC differentiation cultures. Single-cell colony derivation efficiently avoids the risk of reporter silencing in lentiviral insertion. Coupled with pan-CM surface marker such as SIRPA, ventricular CM subtypes (SIRPA+mCherry−) can be also quantified or purified through FACS. Furthermore, combined with the photostable ASAP2f voltage indicator, we generated a dual fluorescent reporter platform that enables sensitive and functional characterization of CM subtypes in beating frequency, voltage fluctuation, or deep action potential analysis. This design not only provides a visible tool to understand the mechanisms underlying electrical conduction between CM subtypes, but also offers tremendous potential for CVD modeling and drug screening.

## Supplementary Information

**Additional file 1: Figure S1.** Routine mycoplasma test of engineered aMHC-mCherry H9 hPSCs. Representative PCR gel image of tested negative control (NC), inhibition control (IC), positive control (PC) and sample (S) were shown.

**Additional file 2: Figure S2.** Immunostaining against MLC2v (A) or MLC2a (B) with day 30 aMHC-mCherry CMs. Scale bar: 100 μm.

**Additional file 3: Figure S3.** Characterization of dual aMHC-mCherry and ASAP2f reporter H9 hPSCs. (A) Representative brightfield (BF) and ASAP2f images as well as flow cytometry analysis of live hPSCs were shown. (B) Representative immunostaining and flow cytometry analysis of NANOG, OCT4, and SSEA4 were shown. Scale bars, 100 μm.

**Additional file 4: Figure S4**. Isoprenaline test with generated CMs from the reporter cell line. Cells were incubated with isoprenaline of indicated concentrations for 5 min at 37°C and taken videos for the following fluorescence analysis. After video collection was complete, the media was changed with fresh culture media.

**Additional file 5: Video S1** Brightfield and mCherry videos of aMHC-mCherry CMs on day 11.

**Additional file 6: Video S2.** Brightfield and mCherry videos of aMHC-mCherry CMs on day 11.

**Additional file 7: Video S3.** Brightfield, ASAP2f and mCherry videos of aMHC-mCherry CMs infected by ASAP2f lentivirus on day 29.

**Additional file 8: Video S4.**Brightfield, ASAP2f and mCherry videos of aMHC-mCherry CMs infected by ASAP2f lentivirus on day 29.

**Additional file 9: Video S5.**Brightfield, ASAP2f and mCherry videos of aMHC-mCherry CMs infected by ASAP2f lentivirus on day 29.

## Data Availability

All data generated or analyzed during this research are included in this published article.

## Abbreviations

CM: Cardiomyocytes
hPSC: Human pluripotent stem cells
CVD: Cardiovascular diseases
FHF: First heart field
aMHC: Alpha myosin heavy chain
GFP: Green fluorescent protein
cTNT: Cardiac troponin T
FACS: Fluorescence-activated cell sorting
