# Supplementary figures and images for: A dual cardiomyocyte reporter model derived from human pluripotent stem cells
Source: Stem Cell Res Ther. 2021 May 29;12:305. doi: 10.1186/s13287-021-02341-6 (PMC8164304; doi:10.1186/s13287-021-02341-6)

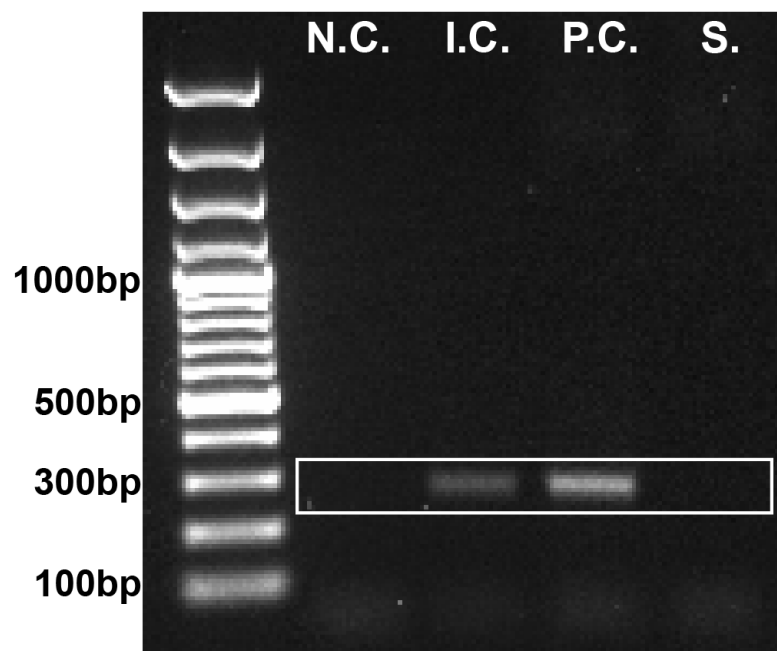

Supplement: Supplementary file 1 — Additional file 1: Figure S1. Routine mycoplasma test of engineered aMHC-mCherry H9 hPSCs. Representative PCR gel image of tested negative control (NC), inhibition control (IC), positive control (PC) and sample (S) were shown. [file 13287_2021_2341_MOESM1_ESM.pdf]

**A**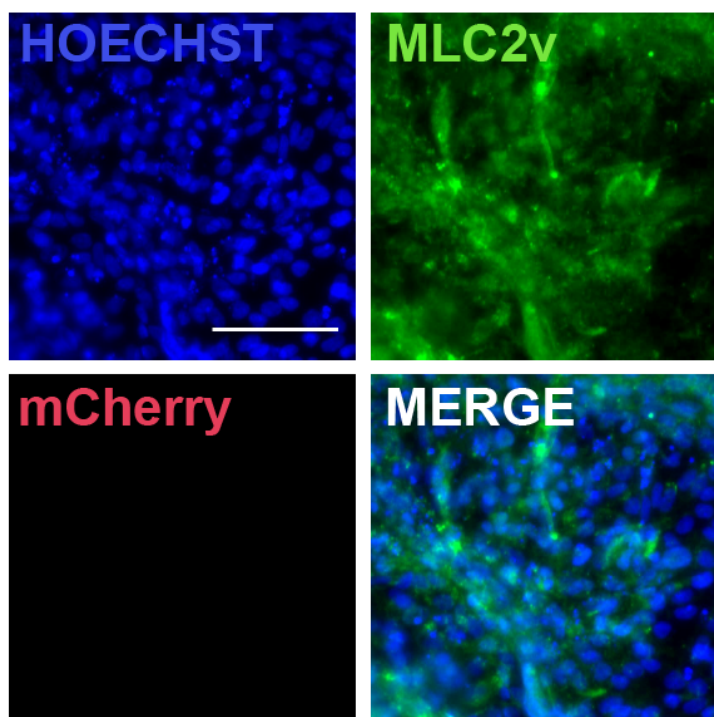**B**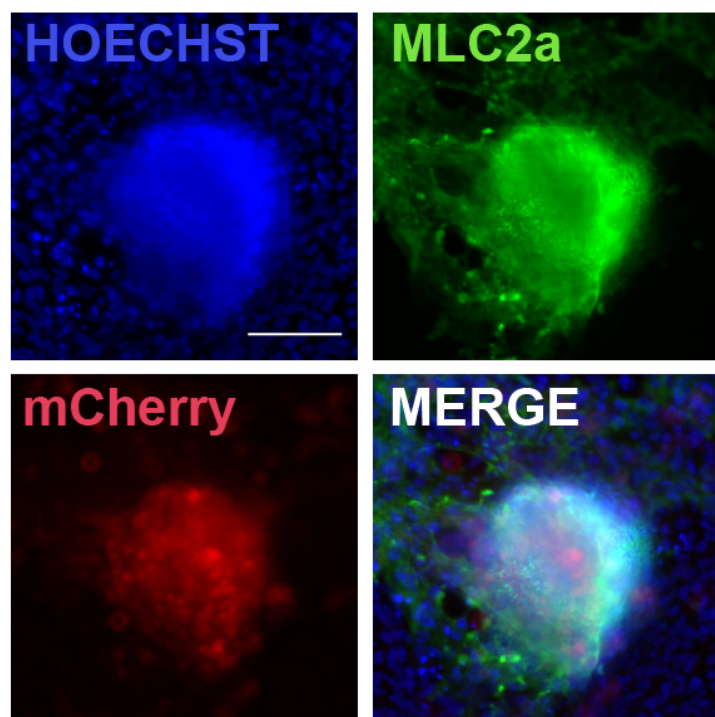

Supplement: Supplementary file 2 — Additional file 2: Figure S2. Immunostaining against MLC2v (A) or MLC2a (B) with day 30 aMHC-mCherry CMs. Scale bar: 100 μm. [file 13287_2021_2341_MOESM2_ESM.pdf]

**A**

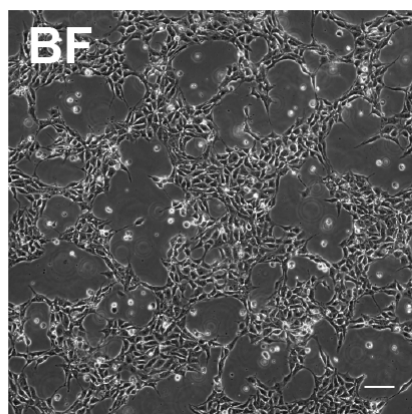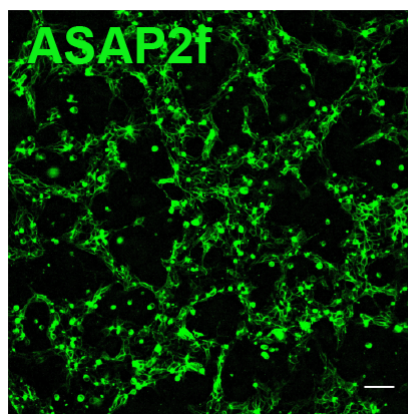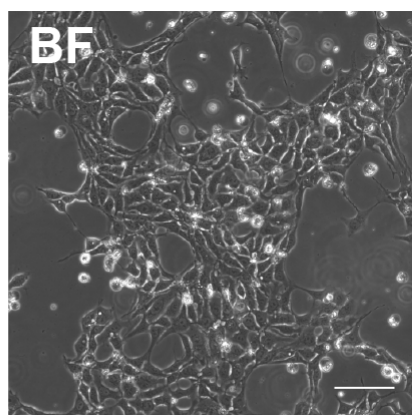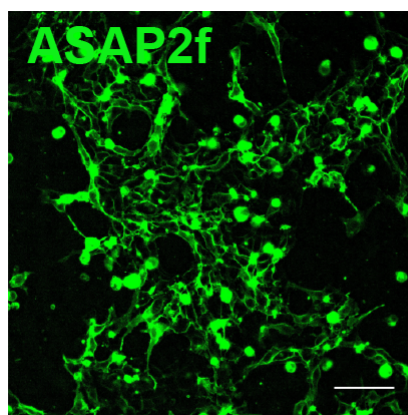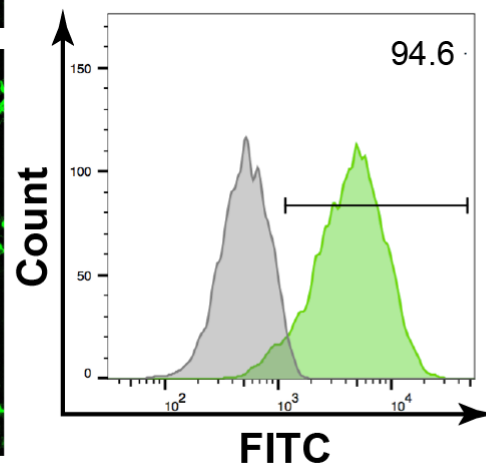

**B**

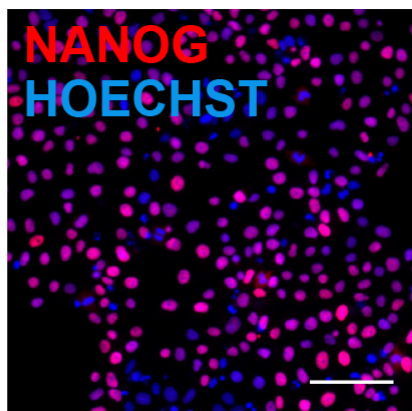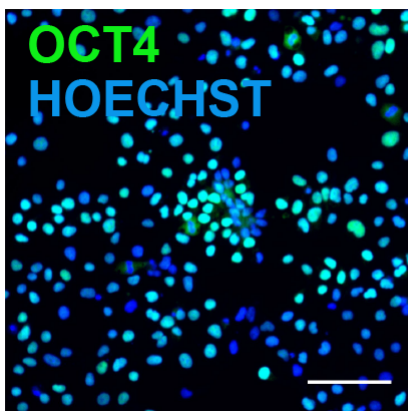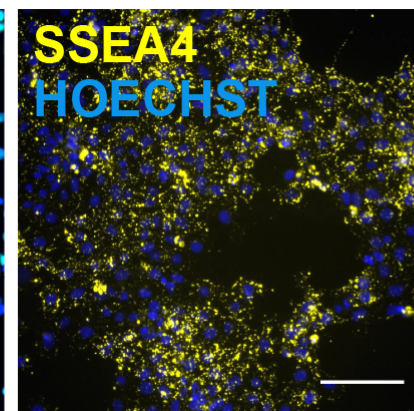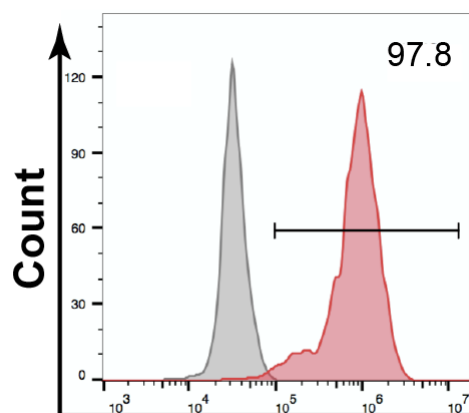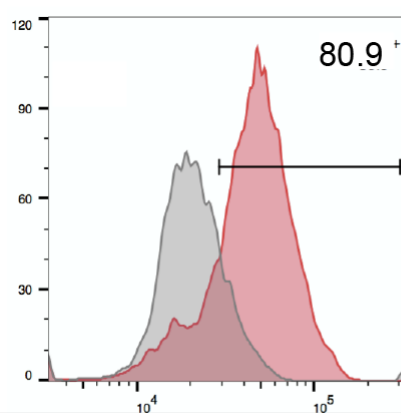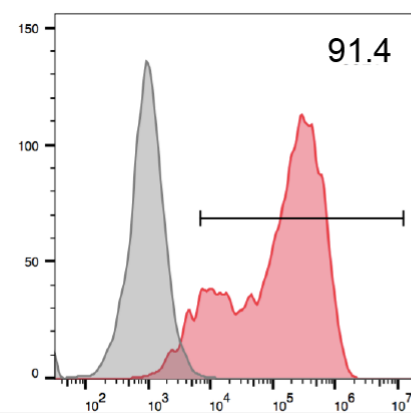

CTRL  
MHC-mcherry H9  
+ASAP2f

**APC**

Supplement: Supplementary file 3 — Additional file 3: Figure S3. Characterization of dual aMHC-mCherry and ASAP2f reporter H9 hPSCs. (A) Representative brightfield (BF) and ASAP2f images as well as flow cytometry analysis of live hPSCs were shown. (B) Representative immunostaining and flow cytometry analysis of NANOG, OCT4, and SSEA4 were shown. Scale bars, 100 μm. [file 13287_2021_2341_MOESM3_ESM.pdf]

**A**

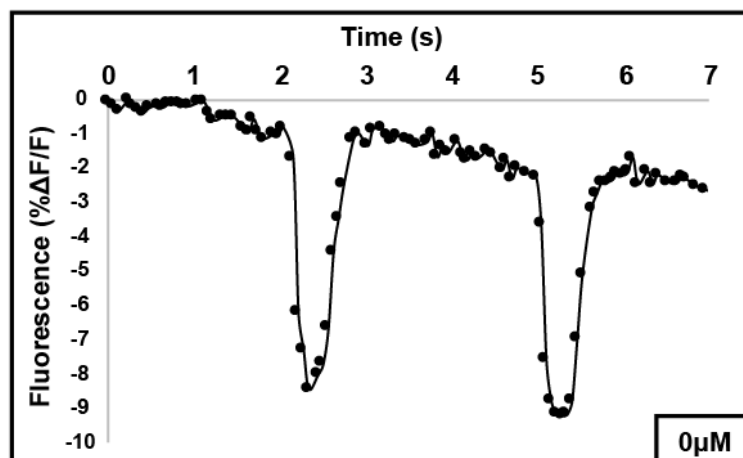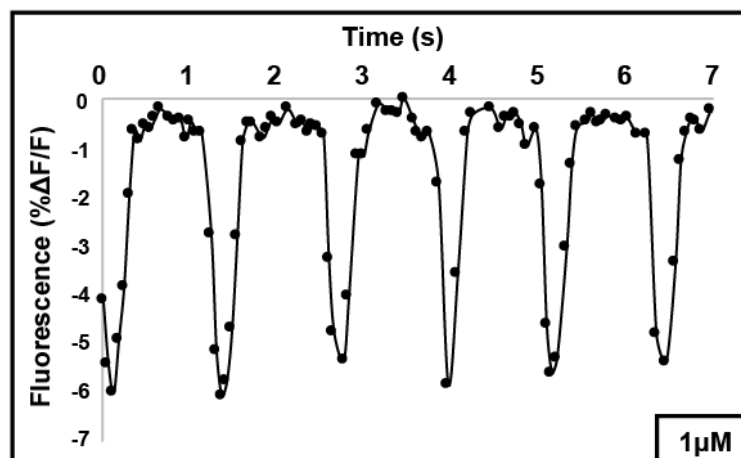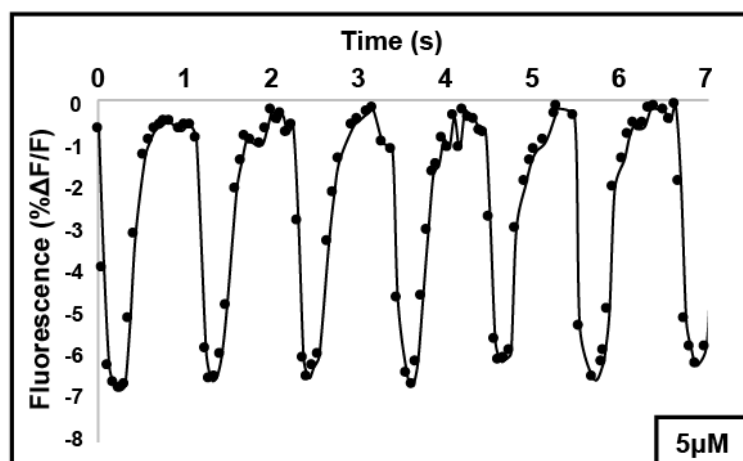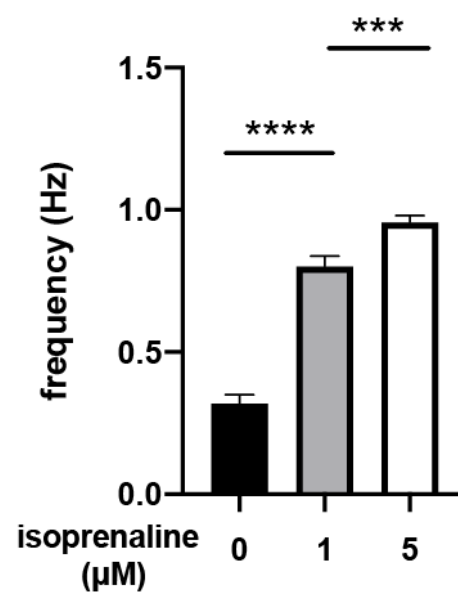

Supplement: Supplementary file 4 — Additional file 4: Figure S4. Isoprenaline test with generated CMs from the reporter cell line. Cells were incubated with isoprenaline of indicated concentrations for 5 min at 37°C and taken videos for the following fluorescence analysis. After video collection was complete, the media was changed with fresh culture media. [file 13287_2021_2341_MOESM4_ESM.pdf]
